# Glucose-Sparing Action of Ketones Boosts Functions Exclusive to Glucose in the Brain

**DOI:** 10.1523/ENEURO.0303-20.2020

**Published:** 2020-12-17

**Authors:** Yuri Zilberter, Tanya Zilberter

**Affiliations:** 1 Institut de Neurosciences des Systèmes, Aix-Marseille Universite, Institut National de la Santé et de la Recherche Médicale Unité Mixte de Recherche 1106, Marseille 13385, France; 2 Infotonic Consultancy, Marseille 13009, France; 3 Institute of Theoretical and Experimental Biophysics, Russian Academy of Sciences, 142290, Pushchino, Russia

**Keywords:** aerobic glycolysis, antioxidant system, glucose metabolism, glycogen, ketogenic diet, ketosis

## Abstract

The ketogenic diet (KD) has been successfully used for a century for treating refractory epilepsy and is currently seen as one of the few viable approaches to the treatment of a plethora of metabolic and neurodegenerative diseases. Empirical evidence notwithstanding, there is still no universal understanding of KD mechanism(s). An important fact is that the brain is capable of using ketone bodies for fuel. Another critical point is that glucose’s functions span beyond its role as an energy substrate, and in most of these functions, glucose is irreplaceable. By acting as a supplementary fuel, ketone bodies may free up glucose for its other crucial and exclusive function. We propose that this glucose-sparing effect of ketone bodies may underlie the effectiveness of KD in epilepsy and major neurodegenerative diseases, which are all characterized by brain glucose hypometabolism.

## Significance Statement

The ketogenic diet (KD) was created in the 1920s as a therapy for refractory epilepsy. Since then, evidence accumulated showing its potential for other major neurodegenerative disorders. The exact mechanism of KD’s protective activity still remains unknown, nonetheless. In the brain, ketone bodies can be used for cellular energy, at least partially substituting glucose as brain fuel. However, glucose has essential functions beyond those of just energy supply that cannot be provided by alternative substrates. We propose that the glucose-sparing effect of ketone bodies may underlie the effectiveness of KD in epilepsy and other major neurodegenerative diseases which are all characterized by brain glucose hypometabolism.

## Introduction

There is no universally accepted definition of ketogenic diets (KDs). Moreover, there is a tendency to relax previous quantitative criteria (Zilberter and Zilberter, 2018) introduced almost a century ago (Wilder and Winter, 1922) that were based on macronutrient composition. Perhaps the most practical notion of whether a diet can be considered to be ketogenic is made by Seyfried (2012): it is “as long as the individual has reduced blood glucose and is producing ketones.” Although protein is included in the ketogenic ratio equation (Shaffer, 1921), glucose, ketone bodies, and their interplay determine the dominating metabolic mode: whether the predominant energy supply is glucose or ketone bodies (Westman et al., 2003). The current data allow us to conclude that the functional interaction between glucose and ketone bodies is not a binary winner-take-all process. Here, we attempt to describe more intricate relationships between them.

## Glycolytic ATP Production

In the case of acute energy demand such as during intense network activity, the brain is able to intensify glycolytic ATP production for rapid supply. Aerobic glycolysis (when glucose is partially converted to lactate in the presence of oxygen, producing two ATP molecules) is disproportionate to the oxygen consumption of glucose utilization when the oxygen delivery is adequate (Dienel and Cruz, 2016). In the resting brain, glycolysis and oxidative phosphorylation rates appear well-matched, indicating nearly complete oxidation of glucose. However, in the activated brain at physiological conditions, e.g., during sensory stimulation and mental testing, aerobic glycolysis has been observed in various brain regions (Dienel and Cruz, 2016; Dienel, 2019a). The cellular contribution to aerobic glycolysis is yet unclear and is a matter of debate (Dienel and Cruz, 2016; Barros et al., 2020). As lactate (in addition to pyruvate) is the end-product of the glycolytic pathway during aerobic glycolysis, it can either be cleared out from the brain or partially consumed as supplementary fuel for neurons (Pellerin and Magistretti, 2012; Dienel and Cruz, 2016). Indeed, aerobic glycolysis as an ATP production mechanism, while low-capacity, is rapid, ensuring fast ATP resupply in neurons where the dynamic demand for energy is highest (Yellen, 2018). The notion of fast ATP synthesis (aerobic glycolysis) is supported by an immediate rise in extracellular lactate and reduction in glucose levels during brief visual stimulation coincident with spiking activity (Li and Freeman, 2015).

Neurons are rich in mitochondria, a major source of ATP (Hall et al., 2012). However, for some of the neuronal functions, aerobic glycolysis may be the preferential method of fuel generation, for instance, for fast axonal vesicle transport (Zala et al., 2013). Although synaptic transmission is a very energy-demanding process, many presynaptic nerve terminals lack mitochondria (Devine and Kittler, 2018; Tourigny et al., 2019), although mitochondria can migrate to and/or ATP can diffuse into the presynaptic boutons during enhanced synaptic activity (Chamberlain and Sheng, 2019; Rossi and Pekkurnaz, 2019). Recent studies indicate that presynaptic transmission is dependent on activity-induced glycolysis (Ashrafi and Ryan, 2017), while presynapses can use ATP produced by both glycolysis and oxidative phosphorylation to sustain neurotransmission (Ashrafi and Ryan, 2017; Chamberlain and Sheng, 2019; Rossi and Pekkurnaz, 2019). Moreover, dendritic spines that receive most excitatory synaptic inputs have been shown to contain no mitochondria (Kasthuri et al., 2015), despite being sites of intense energy utilization. Although some ATP might diffuse to spine heads from nearby dendritic mitochondria, it is conceivable that spines are reliant on glycolysis instead (Kasthuri et al., 2015). Glycolytic ATP alone, with its limited production capacity, is unlikely to be sufficient to power ion pumping required for the maintenance of ion gradient and membrane potential (Hall et al., 2012), but it may nevertheless play an important role in fast refueling. For instance, the preferential role of glycolytic energy supply was demonstrated for Na/K-ATPase in fast-twitch skeletal muscle (Okamoto et al., 2001) and cardiac Purkinje cells (Glitsch and Tappe, 1993). Aerobic glycolysis during KD has not been studied yet. However, unlike glucose, ketones cannot be involved in aerobic glycolysis, and thus they contribute to ATP production via oxidative phosphorylation only (Cunnane et al., 2020).

## The Cytoplasmic Antioxidant System

Reactive oxygen species (ROS) in brain cells originate from multiple sources and most of them are generated as byproducts of metabolic reactions. Intracellular ROS come mainly from NADPH oxidase (NOX), xanthine oxidase, nitric oxide synthase, and mitochondria. NOX is the only enzyme with the primary function of generating ROS (Bedard and Krause, 2007; Koju et al., 2019) that are mostly used for the “host defense” (e.g., microbial killing) in organisms. NOX enzymes are predominantly expressed in the cellular plasma membrane (Ma et al., 2017). The enzyme consists of a membrane-bound catalytic core and several cytosolic regulatory subunits (Bedard and Krause, 2007). There are seven known isoforms of NOX with NOX1, NOX2, and NOX4 expressed in multiple brain regions including the cerebral cortex, hippocampus, cerebellum, hypothalamus, midbrain, and/or striatum (Hou et al., 2020). These NOX variants are the most prominent isoforms detected in a variety of brain cell types (Cahill-Smith and Li, 2014; Rastogi et al., 2016; Hou et al., 2020), with NOX2 the dominant form expressed by microglia, neurons, and astrocytes. Activation of NOX results in an increase of extracellular H_2_O_2_ levels followed by H_2_O_2_ entry into the cells via aquaporins (Bienert and Chaumont, 2014). Under “resting conditions” in the brain, NOX is normally dormant and therefore its contribution to the total cellular ROS production under resting or physiological conditions is not clear (Brown and Borutaite, 2012). In pathology, when NOX is activated by specific stimulation (Rastogi et al., 2016), its cytosolic subunits translocate to the membrane and associate to the functioning complex. Under these conditions, mitochondria and NOX are the major ROS producers (Tarafdar and Pula, 2018; Barua et al., 2019). However, before its diffusion to the cytoplasm, the internal consumption of H_2_O_2_ in mitochondria is much higher than originally anticipated (Munro and Pamenter, 2019) and during oxidative stress, mitochondria may be victims rather than producers of oxidative damage (Gandhi and Abramov, 2012). Indeed, in AD models, the effects of mitochondrial ROS were found to be much smaller compared with those of NOX-produced ROS (Angelova and Abramov, 2018).

In the cytoplasm, an efficient anti-oxidative system based largely on the glycolysis-associated pentose-phosphate pathway strictly regulates ROS levels to avoid any oxidative injury (Bolaños and Almeida, 2010; Franco et al., 2019; Cherkas et al., 2020). Thus, brain cells use glucose as an energy provider as well as the substrate for cytoplasmic ROS detoxification mechanisms. The combination of ROS overproduction with inadequate antioxidant defenses (such as when glucose utilization is inhibited) results in oxidative stress and consequent damages, e.g., neural cell death and neurodegeneration (Avery, 2011; Tarafdar and Pula, 2018).

An extreme example of the importance of glucose-based antioxidant defense for cellular function was reported by us when glucose in artificial CSF (ACSF) was exchanged for pyruvate in hippocampal slices (Malkov et al., 2014, 2019). The substitution resulted in oxidative stress leading to massive network depolarization (analogous to spreading depression; Pietrobon and Moskowitz, 2014) together with a “metabolic collapse.” Importantly, we obtained similar results replacing glucose with other mitochondrial fuels such as lactate or β-hydroxybutyrate (unpublished), indicating again the unique importance of glucose.

KD improves antioxidant defense by stimulating the endogenous antioxidant system. Mild oxidative stress following KD onset drives nuclear translocation of transcription factor Nrf2, leading to increased synthesis of glutathione which is one of the major components of the antioxidant defense (Pinto et al., 2018; Camberos-Luna and Massieu, 2020).

## Glycogen Production in Astrocytes

The major energy reserve in the brain is glycogen, a macromolecular storage form of glucose (Dienel, 2019b). Found mostly in astrocytes (Dienel and Carlson, 2019; neurons can store less significant amounts; Saez et al., 2014), glycogen is a dynamic participant in brain activity and is regulated by neurotransmitters. Dysregulation of glycogen turnover may cause severe consequences, e.g., Lafora disease with progressive neurodegeneration and epilepsy leading to death in early adulthood (Duran et al., 2019). During intense brain activity, glycogen converted to lactate and released from astrocytes can be used by neurons as mitochondrial fuel. Meanwhile, rapid glycogenolytic generation of ATP may be important for astrocytic energy demands (Dienel and Rothman, 2019), and thus glycogenolysis, by reducing the astrocytic requirement for blood-borne glucose, can spare an equivalent amount of glucose for neuronal utilization (Dienel, 2019a; DiNuzzo et al., 2019). The estimated glucose equivalent of glycogen concentration in astrocytes is up to 40–100 mM. Considering the high rate of glycogenolysis, the amount of glucose that can be released via glycogen breakdown is very significant (Dienel, 2019a; DiNuzzo et al., 2019). Unfortunately, glycogen levels in the human brain during KD have yet to be investigated. In rats, one study reported increased inbound glucose (reflecting glycogen content; DeVivo et al., 1978) under KD, while no change in glycogen content was found in another study (Al-Mudallal et al., 1995) or glycogen was decreased (Bough et al., 2006). Carbohydrate intake does not affect brain glycogen content, while in both muscle and liver it does significantly correlate with glycogen levels (Soya et al., 2018).

## Production of Major Neurotransmitters

Glucose fuels *de novo* synthesis of major neurotransmitters. Precursors for glutamate or GABA are synthesized by astrocytes and then transferred to neurons in the glutamine–glutamate/GABA cycle (Dienel, 2019a). The transmitters released during synaptic activity are transported back to astrocytes, where a fraction (∼25%) is degraded with the remainder released and reused by the neurons. The process is very intense, consuming up to 75% of the entire glucose consumption in the cortex (Schousboe et al., 2013; Hertz and Rothman, 2016). Glucose metabolism is required for the synthesis of glutamate from glutamine in glutamatergic neurons (Bak et al., 2006; Lund et al., 2009; Chowdhury et al., 2014; Hertz and Rothman, 2016), but in GABAergic neurons, β-hydroxybutyrate is capable of replacing glucose for GABA production (Hertz and Rothman, 2016).

## Glucose Utilization in the Brain during Ketosis

The KD is an efficient clinical treatment used for over a century to decrease brain hyperexcitability and seizures via a yet unclear mechanism. Carbohydrate limitation in KD led to a popular notion that glycolysis inhibition may partially reproduce the effects of KD. However, this hypothesis ignores the important fact that glycolysis inhibition a priori results in energy deprivation; meanwhile, no energy deficiency occurs under KD [in rats on KD, the brain ATP level was found either unchanged (Al-Mudallal et al., 1996; Bough et al., 2006) or increased (DeVivo et al., 1978; Nakazawa et al., 1983)]. However, do ketones inhibit glycolysis [as, for instance, does 2-deoxy-d-glucose (Pajak et al., 2019) or iodoacetate (Schmidt and Dringen, 2009)] or do they just compete with glucose as mitochondrial fuel? The NMR study of Valente-Silva et al. (2015) is often cited as evidence of glycolysis inhibition. There are a number of important flaws in this study, however: (1) the 400-μm-thick brain slices were superfused with oxygenated ACSF at 3 ml/min rate that results in anoxic conditions within the tissue (Ivanov and Zilberter, 2011), making oxidative phosphorylation impossible; and (2) the authors used 4-AP to induce network activity, while epileptiform activity induced by 4-AP was itself shown to be a strong inhibitor of glycolysis (Malkov et al., 2018). To the best of our knowledge, there are no other reports on ketones’ direct inhibitory action on glycolysis.

There have been many attempts to estimate brain glucose utilization under KD, with variable results. In awake animals, glucose utilization either increased (Yudkoff et al., 2005), did not change (Al-Mudallal et al., 1995) or decreased (Melø et al., 2006), while brain glucose levels either increased (Melø et al., 2006), did not change (DeVivo et al., 1978; Yudkoff et al., 2005) or decreased (Samala et al., 2011). Utilization of the fluorodeoxyglucose (^18^F)-positron emission tomography (FDG-PET) technique in animals requires anesthesia that introduces a brain state quite different from an awake one. Nevertheless, under anesthesia, glucose utilization either increased (Pifferi et al., 2011; Roy et al., 2012), remained unchanged (Yudkoff et al., 2005), or decreased (LaManna et al., 2009; Zhang et al., 2013). The variability of the results may be explained by the presence of confounding factors such as, e.g., the use of anesthesia, significant age difference and the relatively high interindividual variation in plasma ketones during KD (Pifferi et al., 2011; Roy et al., 2012).

Interestingly, in healthy young/middle age humans, cerebral glucose utilization evaluated by arteriovenous difference was decreased under acute hyperketonemia (a blood infusion of β-hydroxybutyrate; Hasselbalch et al., 1996) or following 3 d of fasting (Hasselbalch et al., 1994). Recent studies using the FDG-PET technique confirmed these results reporting decreased brain glucose utilization in healthy humans under KD (Courchesne-Loyer et al., 2017) or acute hyperketonemia (Svart et al., 2018). Importantly and however, similar recordings during ketogenic intervention in humans suffering mild cognitive impairment (Fortier et al., 2019) or mild-moderate Alzheimer’s disease (AD; Croteau et al., 2018b) revealed no change in brain glucose utilization, while brain ketone metabolism was found to be normal (Castellano et al., 2015; Croteau et al., 2018a). Notably, glucose hypometabolism is a hallmark of AD pathogenesis (Caminiti et al., 2018; Gordon et al., 2018; Butterfield and Halliwell, 2019). Indeed, disrupted glucose metabolism associated with oxidative stress is the common feature of major neurodegenerative diseases (Zilberter and Zilberter, 2017; Cunnane et al., 2020; Tang, 2020) and epilepsy (Pearson-Smith and Patel, 2017; Zilberter and Zilberter, 2017; Patel, 2018). For instance, AD pathology occurs well before (up to two decades prior) the onset of clinical symptoms (Caminiti et al., 2018; Gordon et al., 2018; Butterfield and Halliwell, 2019) with dysfunctional glucose metabolism as one earliest manifestation. In human epilepsy, clinical tests using FDG-PET imaging have established that decreased brain glucose utilization during quiescent (interictal) periods is a widely recognized biomarker of epileptogenesis (Sarikaya, 2015; Lotan et al., 2020). Since glucose utilization underlies vital brain functions such as energy supply and antioxidant defense (see above), it is not surprising that disturbances in glucose metabolism can lead to a chain of harmful consequences, and thus likely represent a major underlying cause of disease initiation and progression (Pearson-Smith and Patel, 2017; Zilberter and Zilberter, 2017; Butterfield and Halliwell, 2019). Therefore, as also suggested previously (Cunnane et al., 2016a; Croteau et al., 2018b), it is logical to conclude that in a normal brain, where glucose can fully cover energy needs, the addition of ketones as mitochondrial fuel competitors reduces the need for a part of glucose function and the total glucose utilization goes down. In pathology, when glucose utilization is impaired leading to energy deficiency, ketone bodies compensate at least partially this energy gap, leaving more glucose available for its other vital functions such as mentioned above.

**Figure 1.**
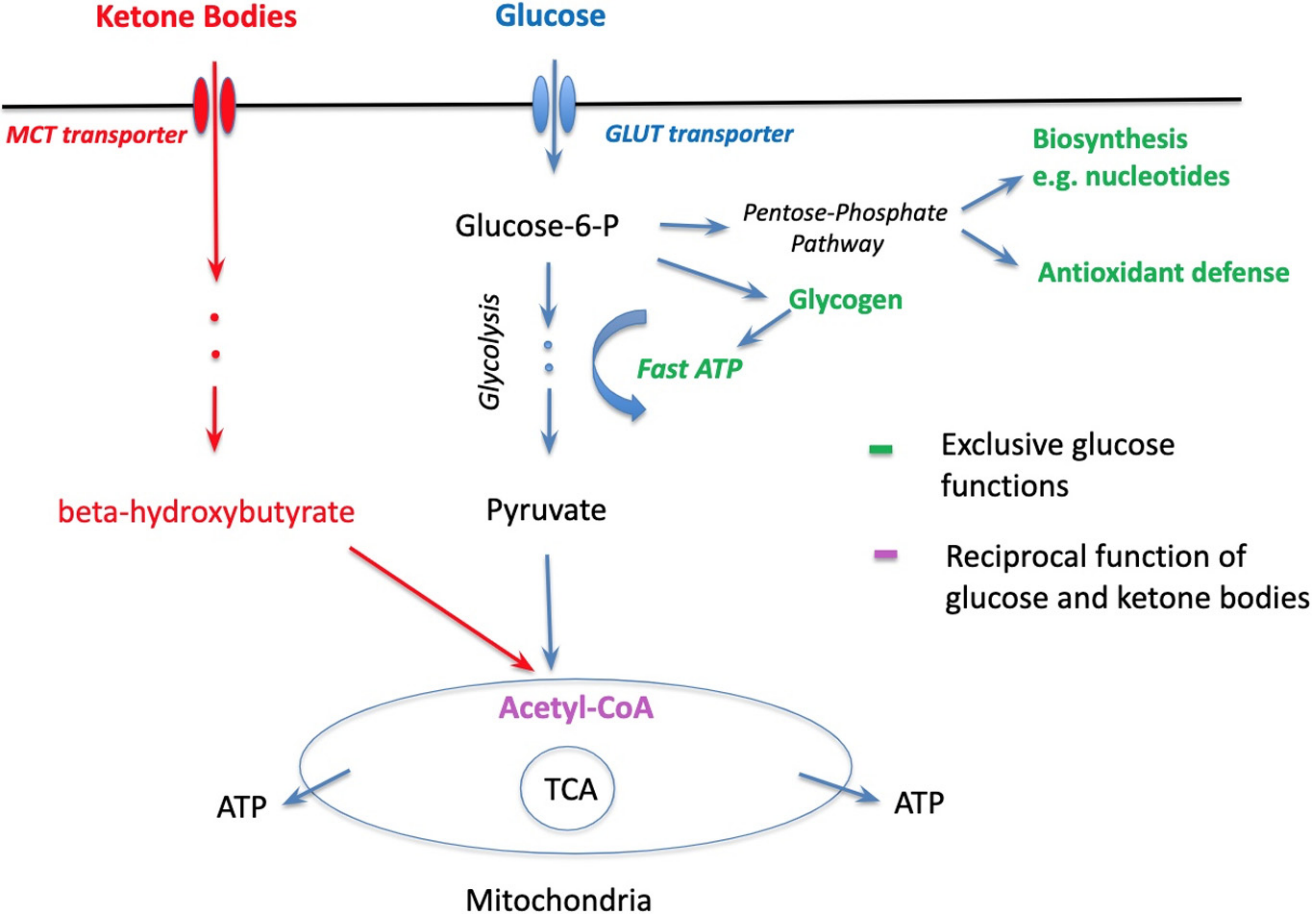
Roles of glucose and ketone bodies in brain cells: parallel, converging, and exclusive. Red: ketone bodies’ pathway to mitochondria. Blue: glucose pathway to mitochondria. Purple: the point of convergence. Green: exclusive roles of glucose.

In fact, the glucose-sparing effect may be dominant in ketone’s beneficial therapeutic function ensemble. Clinical effects of KD are mostly known regarding epilepsy as childhood epilepsy has been successfully treated with KD since the 1920s (Wilder, 1921). Recently, however, the KD began to be used in preclinical studies of other disorders, including neurodegenerative, psychiatric, and brain injury (McDonald and Cervenka, 2018; Camberos-Luna and Massieu, 2020; Kraeuter et al., 2020), demonstrating promising efficiency that has also been confirmed in clinical trials in mild cognitive impairment, AD, and Parkinson’s disease patients (for review, see Cunnane et al., 2020). Meanwhile, it has been noted that the efficacy of KD in an array of disorders with distinct pathophysiologies may indicate shared underlying pathologic mechanisms (Kraeuter et al., 2020). In the case of major neurodegenerative diseases, one such mechanism is glucose hypometabolism (Zilberter and Zilberter, 2017; Cunnane et al., 2020; Tang, 2020). Definitely, impaired glucose metabolism may lead to energy deficiency that ketones can partially compensate by boosting mitochondrial oxidation. Here, it is interesting to note that in the case of early AD stages, neither cerebral oxygen consumption (Hoyer et al., 1988; Hoyer, 1992) nor mitochondrial ability for ketone utilization changed (Castellano et al., 2015; Croteau et al., 2018a), suggesting normal mitochondrial functioning (Cunnane et al., 2016b, 2020), despite pronounced glucose hypometabolism. Indeed, we demonstrated recently in hippocampal slices that amyloid-β (Malkov et al., 2020) as well as epileptiform activity (Malkov et al., 2018) strongly inhibited glycolysis while mitochondrial function remained normal or even upregulated, potentially as a compensatory mechanism. This therefore suggests that glucose metabolism deficiency mostly affects functions not related to oxidative phosphorylation and thus ketones possibly leave more glucose available for other vital functions (Fig. 1) that may be especially important for the therapeutic effects of KD. The potential importance of the glucose-sparing effect of ketones has been noted previously in reports considering neurodegenerative diseases (Cunnane et al., 2016a, 2020).

## Conclusions

Glucose does not share some of its functions in the brain with other metabolic substrates, which makes it an exclusive neurometabolite. The frequent claim that ketones directly inhibit the process of glycolysis is not supported by experimental evidence and seems theoretically unlikely. Moreover, glycolysis inhibition modifies a number of cellular functions that lead to unpredictable variations of network excitability, and in the long-run has been shown to result in epileptogenesis (Samokhina et al., 2017, 2020). In pathology, ketones are capable of partially taking over the glucose’s energy fuel role in mitochondria and presumably spare glucose for its other exclusive functions. Here, we summarized the evidence and offer a non-antagonistic view of the ketone bodies-glucose interplay during metabolic shifting. The glucose-sparing effect of ketone bodies may determine the efficiency of the ketogenic regime and is especially important in epilepsy and major neurodegenerative diseases characterized by significantly impaired brain glucose utilization.

## Synthesis

Reviewing Editor: Michael Michaelides, NIDA-NIH Decisions are customarily a result of the Reviewing Editor and the peer reviewers coming together and discussing their recommendations until a consensus is reached. When revisions are invited, a fact-based synthesis statement explaining their decision and outlining what is needed to prepare a revision will be listed below. The following reviewer(s) agreed to reveal their identity: Leandro Vendruscolo, Corinde Wiers.

Reviewer 1

In this opinion manuscript, the authors address a controversial topic about brain fuel use during ketosis and offer an explanation for a glucose-sparing effect.

The authors offered their opinion about a controversial topic. They suggest that the beneficial effect of ketosis in some neurodegenerative and neurological disorders is related to a glucose-sparing effect so that glucose is available for exclusive roles of glucose when ketone bodies are available as a source of energy. They provide a very clear and informative figure on the roles of glucose and ketone bodies in brain cells. Except for very few typographical errors (e.g., “flow” in page 3, last line) and use of abbreviations that were not spelled out (e.g., “FDG” in page 4), this is a very well-written and timely manuscript. This reviewer has no concerns.

Reviewer 2

The manuscript provides a good overview of the functions of glucose and ketones in metabolism, but often lacks arguments to back up the opinion statements

The authors submitted an opinion piece on ketone bodies supporting glucose as an alternative energy fuel in the brain. Although the topic is of great interest, the manuscript tends to be a bit chaotic at times and arguments made in the manuscript do not always appear to follow from cited work.

Specific comments to the text:

- The significance statement is identical to the abstract.

- The introduction is rather vague and contains hardly any references. E.g., what does it mean that “the dietary landscape is rapidly changing", or “The fat-dominating ketogenic diets (KD) are taking health-conscious populations by storm thanks to social networks and the grassroots movement”?

- The authors provide functions of glucose that are beyond energy production, including (1) glycolytic ATP production, (2) cytoplasmic antioxidant system, and (3) glycogen production in astrocytes. For these three first functions, however, the author provides no mentioning of how these functions occur or change during ketosis. This to me is strange given this is the main topic of the opinion piece.

- For function (4) Production of major neurotransmitters: Could the authors provide a reference for the “fact”: “Glucose metabolism is required for the synthesis of glutamate from glutamine in glutamatergic neurons, but in GABAergic neurons, β-hydroxybutyrate is capable of replacing glucose for this function.”?

- Also, why is the following paragraph added to the paragraph of neurotransmitters? “while measurements in awake rats suggested that only about 50% of active brain’s energy requirements can be supplemented by β-hydroxybutyrate even at saturating plasma concentrations (17mM) -- the rest has to derive from glucose oxidation (Chowdhury et al., 2014). The last conclusion was also confirmed in long-lasting-fasted humans (Owen et al., 1967) and in rats on the ketogenic diet (Zhang et al., 2013a).”

- In the next paragraph, (5) glucose utilization in the brain during ketosis, the authors state “there is no energy deficiency during KD”. How is this argument congruent with the beforementioned paragraph that only 50% of the brain’s energy requirements can be supplemented by ketones?

- Are there important flows or flaws in the Valente-Silva et al study?

- “In awake animals, glucose utilization either did not change (al-Mudallal et al., 1995) or increased (Yudkoff et al., 2005), while brain glucose levels did not change (DeVivo et al., 1978; Yudkoff et al., 2005) or decreased (Samala et al., 2011)”. Yudkoff et al (2015) showed lower plasma glucose levels in KD and argue that “label in 1-13C glucose and 13C lactate were higher in ketotic animals reflecting the fact that the isotope was administered into a peripheral blood glucose pool that is lower in the experimental group.” They did, however, not conclude that brain glucose utilization is higher perse.

- Glucose utilization increased in the study of Roy et al (2012) were performed in aged animals using PET FDG, whereas the other studies cited in the same sentence were done in normally aged rats using PET (LaManna et al, 2009; Zhang et al, 2013b) or using mass spectrometry (Zhang et al, 2013a). Could the authors provide more details to the studies summed up in one sentence? The age and methods used likely very much influence the findings. That is, both PET studies in young rats confirmed decreased glucose uptake in KD.

- Why is a 20% glucose reduction considered small after a 4-day KD? (re: “only”?)

- Why is it “most likely” that ketones’ substitution of glucose in mitochondria spares glucose for vital functions such as antioxidant defense and fast glycolytic ATP production? I don’t see good arguments for this. Which decrease in glucose utilization would you expect for the inhibition of glycolysis?

## Author Response

Response to Reviewers.

Reviewer #1

Thank you very much for the comments. The opinion text has been substantially modified, all changes are in blue.

Reviewer #2

Thank you very much for the valuable comments. The opinion text has been substantially modified, all changes are in blue.

- The significance statement is identical to the abstract.

The significance statement was changed.

- The introduction is rather vague and contains hardly any references. E.g., what does it mean that “the dietary landscape is rapidly changing", or “The fat-dominating ketogenic diets (KD) are taking health-conscious populations by storm thanks to social networks and the grassroots movement”?

The introduction was renewed completely.

- The authors provide functions of glucose that are beyond energy production, including (1) glycolytic ATP production, (2) cytoplasmic antioxidant system, and (3) glycogen production in astrocytes. For these three first functions, however, the author provides no mentioning of how these functions occur or change during ketosis. This to me is strange given this is the main topic of the opinion piece.

We provided short notes considering KD at the end of each function description.

- For function (4) Production of major neurotransmitters: Could the authors provide a reference for the “fact”: “Glucose metabolism is required for the synthesis of glutamate from glutamine in glutamatergic neurons, but in GABAergic neurons, β-hydroxybutyrate is capable of replacing glucose for this function.”?

This issue is discussed in detail in a comprehensive review of Hertz and Rothman (Hertz and Rothman, 2016). In short, both glutamate and GABA are produced from glutamine delivered by astrocytes. In glutamatergic neurons, all glutamate formed from glutamine first enters mitochondria and then is returned to cytosol in a complex process, which requires simultaneous glucose metabolism. In GABAergic neurons, a part of GABA is formed directly in cytosol from glutamine-produced glutamate, while the production of another part from mitochondria-delivered glutamate requires acetyl CoA, which can be supplied by either glucose or β -hydroxybutyrate. This makes the GABA synthesis much less sensitive to the replacement of glucose with

β-hydroxybutyrate than the synthesis of glutamate. The reference is included in the text.

- Also, why is the following paragraph added to the paragraph of neurotransmitters? “while measurements in awake rats suggested that only about 50% of active brain’s energy requirements can be supplemented by β-hydroxybutyrate even at saturating plasma concentrations (17mM) -- the rest has to derive from glucose oxidation (Chowdhury et al., 2014). The last conclusion was also confirmed in long-lasting-fasted humans (Owen et al., 1967) and in rats on the ketogenic diet (Zhang et al., 2013a).”

This text has been removed.

- In the next paragraph, (5) glucose utilization in the brain during ketosis, the authors state “there is no energy deficiency during KD”. How is this argument congruent with the beforementioned paragraph that only 50% of the brain’s energy requirements can be supplemented by ketones?

The references confirming the statement “there is no energy deficiency during KD” are inserted in the new text. Ketones are able to substitute glucose efficiently in mitochondria but they cannot substitute ATP generated in aerobic glycolysis. During KD, however, both ketones and glucose present in the brain that presumably maintains aerobic glycolysis efficient enough.

- Are there important flows or flaws in the Valente-Silva et al study?

Corrected.

- “In awake animals, glucose utilization either did not change (al-Mudallal et al., 1995) or increased (Yudkoff et al., 2005), while brain glucose levels did not change (DeVivo et al., 1978; Yudkoff et al., 2005) or decreased (Samala et al., 2011)”. Yudkoff et al (2015) showed lower plasma glucose levels in KD and argue that “label in 1-13C glucose and 13C lactate were higher in ketotic animals reflecting the fact that the isotope was administered into a peripheral blood glucose pool that is lower in the experimental group.” They did, however, not conclude that brain glucose utilization is higher perse.

We agree with your remark. Note, however, that in Table 3, both brain glucose and lactate are increased in ketotic animals, and the lactate increase is problematic to interpret otherwise than the result of enhanced glycolysis.

- Glucose utilization increased in the study of Roy et al (2012) were performed in aged animals using PET FDG, whereas the other studies cited in the same sentence were done in normally aged rats using PET (LaManna et al, 2009; Zhang et al, 2013b) or using mass spectrometry (Zhang et al, 2013a). Could the authors provide more details to the studies summed up in one sentence? The age and methods used likely very much influence the findings. That is, both PET studies in young rats confirmed decreased glucose uptake in KD.

This is correct and the text has been modified accordingly.

- Why is a 20% glucose reduction considered small after a 4-day KD? (re: “only”?)

Corrected.

- Why is it “most likely” that ketones’ substitution of glucose in mitochondria spares glucose for vital functions such as antioxidant defense and fast glycolytic ATP production? I don’t see good arguments for this. Which decrease in glucose utilization would you expect for the inhibition of glycolysis?

The text has been modified considerably for making our hypothesis more clear and substantiated. We have included the human data indicating that under ketogenic intervention the brain glucose utilization goes down but only in healthy recipients, while it is unchanged in patients with AD pathology characterized by pronounced glucose hypometabolism. Please see the details in the text in blue.
